# Antimicrobial and Immunomodulatory Effects of *Bifidobacterium* Strains: A Review

**DOI:** 10.4014/jmb.2007.07046

**Published:** 2020-10-20

**Authors:** Hyun Jung Lim, Hea Soon Shin

**Affiliations:** College of Pharmacy, Duksung Women’s University, Seoul 01369, Republic of Korea

**Keywords:** Antimicrobial, antiviral, bifidobacteria, probiotics

## Abstract

*Bifidobacterium* strains can provide several health benefits, such as antimicrobial and immunomodulatory effects. Some strains inhibit growth or cell adhesion of pathogenic bacteria, including multidrug-resistant bacteria, and their antibacterial activity can be intensified when combined with certain antibiotics. In addition, some strains of bifidobacteria reduce viral infectivity, leading to less epithelial damage of intestinal tissue, lowering the virus shedding titer, and controlling the release of antiviral substances. Furthermore, bifidobacteria can modulate the immune system by increasing immunoglobulins, and inducing or reducing pro- or antiinflammatory cytokines, respectively. In particular, these anti-inflammatory effects are helpful in the treatment of patients who are already suffering from infection or inflammatory diseases. This review summarizes the antimicrobial effects and mechanisms, and immunomodulatory effects of *Bifidobacterium* strains, suggesting the potential of bifidobacteria as an alternative or complementary treatment option.

## Introduction

Bifidobacteria are gram-positive, strict anaerobic, pleomorphic rods. The name ‘*Bifidobacterium*’ signifies the branching morphology of the bacteria—‘bifidus’ in Latin means cleft in two parts. In addition to their main phenotypic characteristic of producing lactic acid, bifidobacteria produce acetic acid that suppresses the growth of harmful bacteria due to its strong bactericidal action [1, 2].

*Bifidobacterium* was first isolated in 1899 from the feces of healthy breast-fed infants by Tissier of the Pasteur Institute in France. At birth, many bacterial species gain access into the intestinal tract of infants; however, bifidobacteria gradually become established as the main bacteria, and predominate in the intestinal microflora during the neonatal period, especially in breast-fed infants. As the intestinal microflora changes with age, there is an accompanying reduction in the percentage of bifidobacteria [3, 4].

Amongst approximately 1000 species of bacteria in the gut, harmful bacteria are those which possess pathogenicity or transform food components into harmful substances [5]. A favorable intestinal microflora consists of a low level of harmful bacteria and a high level of beneficial bacteria, the latter represented by bifidobacteria. In the large intestine, the number of *Lactobacillus* is approximately 1% that of *Bifidobacterium* [6].

Bifidobacteria help in maintaining the intestinal microbial balance owing to their potentially beneficial physiological effects. Bifidobacteria are ingested as probiotics to improve the intestinal microflora balance. This, in turn, improves the intestinal environment and contributes to the overall health of the intestine, with the ultimate aim to prevent or treat intestinal tract diseases or infection. In addition, studies have shown that bifidobacteria enhance several immune response processes, including promoting the involvement of macrophages and lymphocytes.

Here, we provide a broad review of several *Bifidobacterium* strains as the most promising probiotic species, and discuss their antibacterial, antiviral, anti-inflammatory, and pro-inflammatory effects. This review also examines recent studies on several *Bifidobacterium* strains that investigated their antimicrobial mechanisms and immunomodulatory effects in certain disease conditions.

## Antimicrobial Effects

### Antibacterial Effects

Several studies have shown that a number of *Bifidobacterium* strains have antibacterial activity against various pathogens, including *Escherichia coli*, *Salmonella typhi* [7], *Streptococcus mutans* [8], *Propionibacterium acnes* [9], and *Listeria monocytogenes* [10]. Moreover, some studies have reported that *Bifidobacterium* strains can be used against multidrug-resistant pathogens. One study showed that probiotics, including some *Lactobacillus* strains, could inhibit or remove the biofilm formed by multidrug-resistant *E. coli*; amongst all the tested probiotics, *B. longum* showed the strongest inhibition against the biofilm formed by *E. coli* IC2 [11].

*Bifidobacterium* strains, such as *B. adolescentis*, *B. pseudocatenulatum*, and *B. longum*, were found to inhibit the growth of vancomycin-resistant *S. aureus* and vancomycin-resistant *Enterococcus* [12]. The cytoprotective effects of bifidobacteria, and their activity against multidrug-resistant *S. aureus* (MRSA), were investigated in mice treated with multiple antibiotics and then infected with MRSA, followed by treatment with 5-fluorouracil. The results showed that the antibacterial effect was more pronounced when *B. breve* and galactooligosaccharides were combined as synbiotics, compared to *B. breve* alone [13]. Another study by Yang et al. showed that the antibacterial activity of *B. breve* against *Clostridium difficile* was enhanced when combined with certain antibiotics, such as metronidazole, clindamycin, and ceftazidime [14]. Oral administration of *B. longum* with *L. rhamnosus* increased the concentration percentage of *B. longum* in the intestine, and the effect lasted for one month after the end of probiotic intake. In addition, the intestinal microflora composition was affected at the phyla and species levels, resulting in a reduction in harmful bacteria, such as *Ruminococcus gnavus* (which possess different pathogenic traits and virulence factors), and an increase in beneficial bacteria, including *Akkermansia muciniphila,* which is closely related to human health and inversely associated with body fat mass and glucose intolerance (Table 1) [15].

### Antiviral Effects

Many studies investigating the antiviral effects of bifidobacteria have focused on their activity against rotavirus, one of the most important pathogens that causes diarrhea and dehydration in children, resulting in numerous deaths in developing countries [16]. In an in vitro study, the administration of *B. thermophilum* inhibited rotaviral adherence to Caco-2 and HT-29 cells, and maintained the viral titer at a low level. In another study, pretreatment with *B. thermophilum* led to less epithelial damage of intestinal tissue, followed by its recovery to a normal state [17]. Similar results were observed when porcine intestinal epitheliocytes were pretreated with *B. infantis* or *B. breve* [18].

The antiviral effects of bifidobacteria were demonstrated in several in vivo studies as well. In mouse models, a lower virus shedding titer and shorter diarrhea period were observed, and these protective effects were more evident in colonized and vaccinated neonatal gnotobiotic pigs than in non-colonized and vaccinated neonatal gnotobiotic pigs [19]. In another study, oral administration of *B. infantis* led to an approximate 67% increase in fecal secretory IgA [20]. In a randomized and controlled trial, breast-fed infants had higher fecal secretory immunoglobulin A (sIgA) concentrations than formula-fed infants, and the fecal sIgA concentration increased with *B. lactis* consumption. Likewise, anti-rotaviral and anti-polioviral sIgA increased after *B. lactis* consumption in cesarean-delivered infants, suggesting that the immune system of formula-fed and cesarean-delivered infants can be improved by the intake of bifidobacteria (Table 2) [21].

### Antimicrobial Mechanisms

As described above, numerous studies have confirmed the antibacterial and antiviral activities of probiotics, including those of *Bifidobacterium* strains. The mechanisms of these effects were investigated mostly in vitro. In a study conducted to confirm whether the viability of bifidobacteria determined their adhesion to the cell, various enzymes and chemical compounds were treated with *B. animalis*. The results showed that a substance on the outer layer of the cell wall, and not the viability of the cell, affected the adherence ability of bifidobacteria. It was observed that, while treatment with digestive enzymes reduced the adhesion ability of bifidobacteria, it did not significantly affect cell viability. Furthermore, sodium metaperiodate treatment completely killed the bacteria, but their adhesion ability increased [22]. With regards to gene expression, the expression of REGIII-γ (a murine antibacterial peptide that is expressed more effectively when the microflora is more diverse) was induced after the inoculation of *B. breve*. Similarly, the expression of REGIII-α, a human homolog of REGIII-γ, was induced when live and heat-inactivated *B. breve* were inoculated into Caco-2 cells [23]. This reflected strengthening of the mucous membrane and the protective effects of *B. breve* against infection and inflammation.

**Fig. 1:**
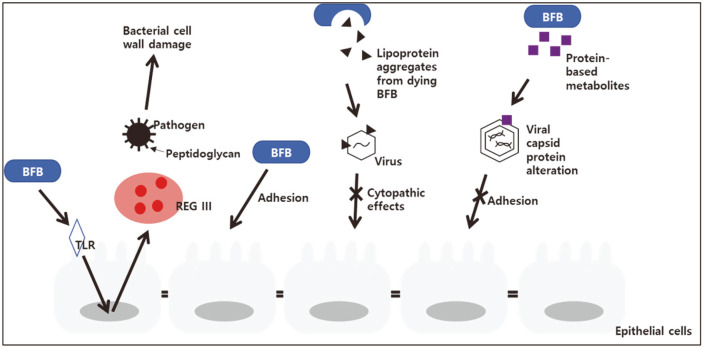
Antimicrobial mechanisms of bifidobacteria.

A study investigating the mechanism of the antiviral effect of bifidobacteria showed that, when *B. longum* was cultured for 72 h, the number of bacteria decreased but they were still effective in protecting cells against coxsackievirus-induced cytopathogenicity. This effect was attributed to an increase in the concentration of a compound on the cell walls of the dying bifidobacteria. Further investigation revealed that the compound was lipoprotein A, which combined with a viral capsid protein, the coxsackievirus and adenovirus receptor. Interestingly, the protective effect was observed when bifidobacteria were pre-incubated with the virus, but not with HEp-2 cells [24]. Similar results were obtained in a study in which the anti-rotaviral activity of bifidobacteria was evaluated in MA104 cells. When probiotic metabolites were pre-incubated with rotavirus, infectivity of the cells was reduced. In this case, the single metabolite that significantly reduced viral infections was a protein-based metabolite extracted from *B. adolescentis* (Table 3, Fig. 1) [25].

## Immunomodulatory Effects

### Pro-inflammatory Effects

In a trial involving nondiabetic subjects, the serum levels of pro-inflammatory cytokines (*i.e.*, interleukin (IL)-12, interferon-γ (IFN-γ), and immunoglobulin 1) increased significantly in the test group that was fed dairy yogurt containing *L. paracasei*, *B. lactis* and heat-killed *L. plantarum*. In addition, there was an increase in the activity of natural killer cells. The results suggested that eating dairy products can help enhance immune responses [26]. In another study, IL-1β, tumor necrosis factor-α (TNF-α), and nitrous oxide were released additively when *Bifidobacterium* strains were treated with lipopolysaccharide (LPS). Furthermore, the presentation of antigens by dendritic cells increased, and cells treated with the supernatant of bifidobacteria were larger and rougher than those exposed to the medium or LPS alone [27]. In a study using a colonic explant, transcription of IL-1α and IL-1β increased when *B. bifidum* was added to the medium. In that study, expression of the polymeric immunoglobulin receptor, responsible for the export of dimeric/polymeric IgA across the intestinal epithelium and contributing to increased sIgA secretion, increased in a concentration-dependent manner [28]. This implied an increase in toll-like receptor signaling (Table 4).

### Anti-Inflammatory Effects

The concentration of TNF-α increased in the colons of mice with dextran sodium sulfate-induced colitis. However, the administration of *B. lactis* reversed this effect, which led to a reduction in intestinal epithelial cell apoptosis. In addition, the administration of *B. lactis* improved pathological properties, such as shortening of colon length, induced by dextran sodium sulfate [29].

In another study, when HT-29 cells were pre-incubated with conditioned media (CM) of intestinal *Bifidobacterium* strains (isolated from healthy infants), the production of IL-8 in response to TNF-α and *E. coli* 055:B5 LPS was significantly inhibited. In particular, CM of all *B. bifidum* strains (except Bif2) used in this study significantly inhibited IL-8 production by LPS. The transcription of transforming growth factor-β1 (TGF-β1; an anti-inflammatory cytokine) and expression of p21CIP (a cyclin-dependent kinase suppressor) increased when the mice were treated with CM and LPS or TNF-α. Although the active substance was not defined, it was not a protein or nucleic acid, because the anti-inflammatory activity was maintained when the CM was treated with proteinase K or a DNase/Rnase mix (after ultrafiltration using a 9 kDa cut-off filter) [30]. Further, in a study using RAW 264.7 macrophages, the concentrations of LPS and TNF-α decreased in all the tested *Bifidobacterium* strains (*i.e.*, *B. bifidum*, *B. longum*, and *B. longum* subsp. *Infantis*). In addition, the concentration of another pro-inflammatory cytokine, IL-1β, decreased in *B. bifidum* (Table 5) [31].

### Immunomodulatory Effects

In a study using peripheral blood mononuclear cells isolated from the venous blood of ulcerative colitis (UC) patients, two ultraviolet irradiation-killed probiotic strains, *L. acidophilus* and *B. lactis*, increased the levels of pro-inflammatory (IFN-γ and TNF-α) and anti-inflammatory (IL-10 and TGF-β) cytokines when compared to those in the control group (peripheral blood mononuclear cells incubated with RPMI-1640 medium). In particular, the levels of IL-10 and TGF-β were relatively higher, while the levels of IFN-γ and TNF-α appeared to be lower in the *B. lactis* group. The levels of IFN-γ and TNF-α were lower at 72 h compared to those at 48 h, which could be due to the increase in IL-10 and TGF-β levels. Thus, it was concluded that *B. lactis* can be administered as an alternative treatment in UC patients suffering from inflammatory responses because it has a more significant effect on the activation of T-regulatory cells and less effect on the activation of T-helper cells, when compared to *L. acidophilus* [32].

In another in vitro study using keratinocytes and fibroblasts incubated with inactivated *S. aureus*, the concentration of IL-18 increased after treatment with inactivated *B. breve* and *B. pseudolongum*. Furthermore, the levels of IL-8 decreased in all inactivated bacteria and live *B. longum* and *B. pseudolongum*, and the decrease was greater in the inactivated bacteria. In the case of IL-6, an anti-inflammatory cytokine, all tested inactivated and live bifidobacteria reduced its levels, with the exception of live *B. pseudolongum* 119^1A^ [33].

**Fig. 2:**
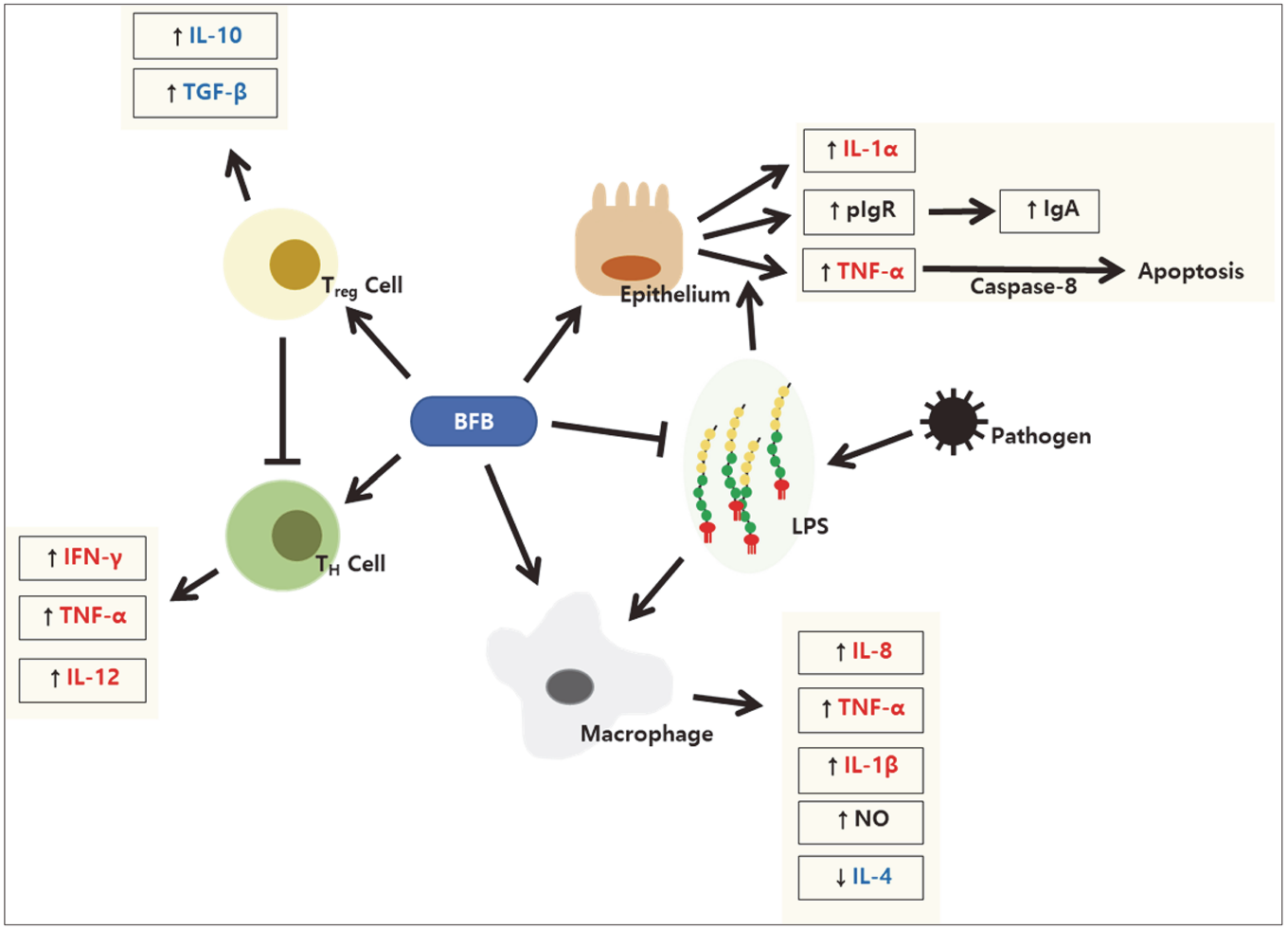
Immunomodulatory mechanisms of bifidobacteria.

In an in vivo experiment using mice infected with *Klebsiella pneumoniae*, the number of inflammatory cells in the lungs was reduced, and the concentration of pro-inflammatory cytokines (TNF-α and IL-6) in bronchoalveolar fluid (which increased after infection with *K. pneumoniae*) was also significantly reduced, when live or inactivated *B. longum* 5^1A^ was administered. Moreover, when live *B. longum* 5^1A^ was administered, production of the anti-inflammatory cytokine, IL-10, was induced in the liver at a significantly higher level compared to that in the control group. In contrast, similar results were not observed with inactivated *B. longum*, suggesting that substances produced by live bacteria induce IL-10 production; this substance was later revealed as acetate. On the other hand, the concentration of a chemokine (CXCL1) increased when live *B. longum* 5^1A^ was administered to germ-free mice, and both live and inactivated *B. longum* 5^1A^ induced reactive oxygen species production in alveolar macrophages, resulting in the elimination of *K. pneumoniae* (Table 6, Fig. 2) [34].

## Conclusions

*Bifidobacterium* strains show antibacterial effects against various pathogens, and some inhibit multidrug-resistant pathogens. In addition, some strains exhibit antiviral effects by inhibiting adherence of the virus to the cells and lowering the viral titer, both in vitro and in vivo. Substances, such as lipoprotein A, found on the outer layer of bifidobacterial cell walls, inhibit pathogens and, in some cases, bifidobacteria induce the expression of antimicrobial substances, such as REGIII, in epithelial cells. Bifidobacteria show both pro- and anti-inflammatory effects. Studies have demonstrated that the expression of inflammatory cytokines, including TNF-α and IL-1, is increased and the activity of natural killer cells is promoted by bifidobacteria, suggesting their potential therapeutic effect in immunosuppressed patients. Other findings show that bifidobacteria inhibit rotaviral adherence leading to less damage of intestinal epithelial tissue. Furthermore, there is a lower virus shedding titer and shorter diarrhea period, along with an increase in sIgA. Additionally, some strains show anti-inflammatory effects by lowering the levels of TNF-α, IL-8, and IL-1β. These effects are particularly helpful in the treatment of inflammatory diseases, including colitis or LPS-induced conditions. In the cases of UC or *S. aureus* and *K. pneumoniae* infections, bifidobacteria modulate the immune system by inducing or suppressing both pro- and anti-inflammatory cytokines. Overall, these results suggest the effectiveness of *Bifidobacterium* strains as potential therapies for infectious or inflammatory diseases. In order for *Bifidobcterium* strains to be applied for pharmaceutical uses, further in vivo studies should be conducted to ensure effectivity and safety of bifidobacterial products in human bodies.

## Figures and Tables

**Table 1:** Antibacterial effects of *Bifidobacterium* spp.

Reference	Strain(s)	Outcomes and results	Discussion
Inturri *et al*. [7]	*B. longum*, *L. rhamnosus*	• Both strains inhibited pathogens (*E. coli*, *S. enteritidis*, and *S. typhi*), both alone and in combination • Inhibited attachment of pathogenic bacteria to HT-29 cells	Probiotics inhibited gram-negative pathogens, and the inhibitory effect was stronger in combination
Lee *et al*. [8]	*B. adolescentis*, *B. longum*	• Proliferation of *S. mutans, S. sobrinus, S. gordoni*, and *Aggregatibacter actinomycetemcomtans* decreased • Live bacteria showed stronger activity	The administration of *B. adolescentis* may be useful in preventing cavities
Lee *et al*. [9]	*B. adolescentis*, *B. pseudo-catenulatum*, *B. longum*	• Antibacterial activity against *Propionibacterium acnes*, *S. aureus*, *S. epidermidis,* and VISA increased	*Bifidobacterium* spp. inhibited the growth of *P. acnes* and thus could be used to treat acne
Muñoz-Quezada *et al*. [10]	*L. paracasei*, *L. rhamnosus*, *B. breve*	• Antibacterial effect against *Listeria monocytogenes* increased	Probiotics were useful in preventing meningitis caused by *L. monocytogenes*
Abdelhamid *et al*. [11]	*L. acidophilus*, *L. rhamnosus*, *B. longum*, *B. bifidum*	• Biofilm formation of *E. coli* IC2 and WW1 was inhibited	Probiotics removed the biofilm formed by multidrug-resistant *E. coli*
Yoon *et al*. [12]	*B. adolescentis*, *B. pseudo-catenulatum*, *B. longum*	• Growth inhibition activity against VISA and VRE increased	Bifidobacteria were beneficial when administered in combination with antibiotics, in diseases caused by superbacteria
Lkhagvadorj *et al*. [13]	*B. breve*, *B. dentium*, *B. pseudo-catenulatum*	• Survival of cells treated with 5-FU and then infected with MRSA increased	The combination of *B. breve* and galactooligosaccharides exhibited activity against MRSA
Yang *et al*. [14]	*B. breve*	• Expression of *tcdA* and *tcdB* decreased when combined with antibiotics, including metronidazole, clindamycin, and ceftazidime	The antibacterial effects of *C. difficile* were enhanced when *B. breve* was combined with certain antibiotics
Toscano *et al*. [15]	*L. rhamnosus*, *B. longum*	• Changes in intestinal microbial composition • Harmful bacteria decreased	Probiotics changed the intestinal microflora by reducing harmful bacteria and increasing beneficial bacteria

5-FU, 5-fluorouracil; MRSA, multidrug-resistant *S. aureus*; VISA, vancomycin-resistant *S. aureus*; VRE, vancomycin-resistant *Enterococcus*.

**Table 2:** Antiviral effects of *Bifidobacterium* spp.

Reference	Strain(s)	Outcomes and results	Discussion
Gagnon *et al*. [17]	*B. thermophilum*, *B. thermacido-philum*, *B. longum*, *B. pseudolongum*	• Inhibition of adherence of rotavirus to Caco-2 and HT-29 cells after pretreatment with *B. thermophilum* • Number of rotavirus, duration of diarrhea, and epithelial lesion decreased after treatment with *B. thermophilum*	Bifidobacteria contributed to the inhibition of rotavirus infections, and ultimately resulted in reduced transmission
Ishizuka *et al*. [18]	*B. infantis*, *B. breve*	• Controlled release of antiviral substances • Rotaviral infectivity of PIE cells decreased with *B. infantis* or *B. breve* pretreatment	It is possible to replace antiviral drugs with a bifidobacteria formula to inhibit rotavirus infections in animals
Vlasova *et al*. [19]	*L. rhamnosus*, *B. animalis*	• Virus shedding titer decreased • Viral diarrhea period reduced	Diarrhea of neonatal gnotobiotic pig by human rotavirus was mitigated
Muñoz *et al*. [20]	*B. longum* subsp. *infantis*	• Virus shedding decreased • Fecal sIgA increased	*B. infantis* showed an initial protective effect against infection with the murine rotavirus McN strain
Holscher *et al*. [21]	*B. lactis*	• Fecal anti-rotaviral and anti-polioviral IgA increased after vaccination	The lack of immunity in infants not breastfed or delivered by cesarean sectioning was mitigated by safely introducing immune-controlling bacteria through a Bb12 formula

IgA, immunoglobulin A; PIE, porcine intestinal epitheliocytes.

**Table 3:** Antimicrobial mechanisms of *Bifidobacterium* spp.

References	Strain(s)	Outcomes and results	Discussion
Wang *et al*. [22]	*B. animalis*	• Adhesion was reduced without any change in viability when treated with enzymes • Viability was reduced but adhesion maintained when treated with lithium chloride • Viability was reduced to zero but adhesion increased when treated with sodium metaperiodate	The adhesion of bifidobacteria was determined by the substance on the outer layer of the cell wall, and not bacterial cell viability
Natividad *et al*. [23]	*B. breve*	• Expression of the REGIII-γ antibacterial peptide increased • Expression of REGIII-α, a human homolog of REGIII-γ, increased	A correlation existed between the expression of REGIII-γ in the colon and the composition of microflora. *B. breve* effectively induced REGIII expression
El Kfoury *et al*. [24]	*B. longum*, *B. breve*	• Protective effect against coxsackievirus increased when bifidobacteria was cultured with viral particles • Damage to HEp-2 cells by CV-B4 E2 was inhibited when CV-B4 was preincubated with LpA of bifidobacteria	The LpA-derived protein of bifidobacteria prevented CV-B4 infection. This effect was attributed to the interaction of the protein with a viral capsid protein in CAR
Fernandez-Duarte *et al*. [25]	*B. adolescentis*, *L. casei*, *L. fermentum*, *B. bifidum*	• No difference in rotavirus infectivity when pretreated with probiotic metabolites • Challenge after co-incubation of metabolites with virus; infectivity decreased	Metabolites of *L. casei* and *B. adolescentis* transformed the rotavirus protein, thus reducing its ability to effectively attach to MA104 cells

CAR, coxsackievirus and adenovirus receptor; LpAs, lipoprotein aggregates.

**Table 4:** Pro-inflammatory effects of *Bifidobacterium* spp.

References	Strain(s)	Outcomes and results	Discussion
Lee *et al*. [26]	*L. paracasei* ssp. *paracasei*, *B. animalis* spp. *lactis*, *L. plantarum*	• TNF-α, IFN-γ, IL-12, and IgG1 increased	*L. paracasei*, *B. lactis*, and heated *L. plantarum* can play key roles in enhancing the immunity of immunosuppressed patients
Han *et al*. [27]	*B. pseudocatenulatum*, *B. longum*, *B. breve*	• External antigen ovalbumin increased • NO release increased • IL-1β and TNF-α increased • Macrophages were larger and rougher	Bifidobacteria activated macrophages and enhanced APC cell functions through MHC class molecules, and increased the secretion of macrophage intermediates
Nakamura *et al*. [28]	*B. bifidum*	• pIgR mRNA increased • Expression of IL-1α and IL-1β increased	Expression of intestinal pIgR increased site-specifically. This can be explained by the action of epithelial cells through TLRs

APC, antigen presenting cell; IL-1β, interleukin 1 beta; MHC, major histocompatibility complex; NO, nitric oxide; pIgR, polymeric immunoglobulin receptor; TLRs, toll-like receptors; TNF-α, tumor necrosis factor-α.

**Table 5:** Anti-inflammatory effects of *Bifidobacterium* spp.

References	Strain(s)	Outcomes and results	Discussion
Chae *et al*. [29]	*B. animalis* subsp. *Lactis*	• Shortening of colon length reduced • Pathological properties of colon by DSS treatment decreased • DSS-induced apoptosis of intestinal epithelial cell decreased • Increased TNF-α of DSS-treated mouse reduced	BB12 lowered the sensitivity to colitis, induced by DSS, and reduced the apoptosis of intestinal epithelial cells. This was attributed to the reduction in TNF-α
Khokhlova *et al*. [30]	*B. bifidum*, *B. longum*, *B. breve*, *B. adolescentis*	• Inhibited IL-8 secretion of LPS-treated HT-29 cells (conditioned medium of all tested strains)	Bifidobacteria controlled the signaling pathway in epithelial cells and was species-specific
Rodes *et al*. [31]	*L. rhamnosus*, *B. bifidum*, *B. longum*, *B. longum* subsp. *Infantis*	• Concentration of LPS decreased • TNF-α and IL-1β decreased in intestinal LPS	Probiotics lowered the intestinal LPS concentration and reduced the secretion of pro-inflammatory cytokines in macrophages

DSS, dextran sodium sulfate; IL-8, interleukin 8; IL-1β, interleukin 1 beta; LPS, lipopolysaccharide; TNF-α, tumor necrosis factor-α.

**Table 6:** Immunomodulatory mechanism of *Bifidobacterium* spp.

References	Strain(s)	Outcomes and results	Discussion
Sheikhi *et al*. [32]	*L. acidophilus*, *B. lactis*	• IL-10, TGF-β, IFN-γ, and TNF-α increased compared to control in both probiotic groups • IL-10, TGF-β were relatively higher in *B. lactis* group • IFN-γ, TNF-α appeared to be relatively lower in the *B. lactis* group	*B. lactis* activated not only Th, but also Treg cells. It can be used as a treatment alternative for UC patients
Silva *et al*. [33]	*B. longum*, *B. breve*, *B. pseudolongum*, *B. bifidum*	• Bacterial growth of all strains was inhibited • Decrease in IL-8 was greater with live *B. longum* • IL-6 decreased in all live bacteria	*B. longum* showed antibacterial activity, and *B. bifidum* and *B. bifidum* were effective in producing cytokines and extracellular matrix
Vieira *et al*. [34]	*B. longum*	• TNF-α and IL-6, which were increased after *K. pneumoniae* infection in bronchoalveolar fluid, decreased • Increased IL-10 with live *B. longum* • Increased ROS production with greater effect of live bacteria	Live *B. longum* probiotics can activate the TLR signal pathway to generate ROS, regulate the inflammatory response, and help the lungs quickly restore constancy

IL, interleukin; ROS, reactive oxygen species; TGF, transforming growth factor; Th, T-helper cells; Treg, T-regulatory cells; TLR, toll-like receptor; TNF-α, tumor necrosis factor-α; UC, ulcerative colitis.
